# Description-based reappraisal regulate the emotion induced by erotic and neutral images in a Chinese population

**DOI:** 10.3389/fnhum.2012.00355

**Published:** 2013-01-10

**Authors:** Jiaxin Peng, Chen Qu, Ruolei Gu, Yue-Jia Luo

**Affiliations:** ^1^State Key Laboratory of Cognitive Neuroscience and Learning, Beijing Normal UniversityBeijing, China; ^2^Center for Studies of Psychological Application, South China Normal UniversityGuangdong, China; ^3^Key Laboratory of Behavioral Science, Institute of Psychology, Chinese Academy of SciencesBeijing, China; ^4^Sichuan Research Center of Applied Psychology, Chengdu Medical CollegeChengdu, China

**Keywords:** LPP, SW, up-regulation, emotion regulation, description-based reappraisal

## Abstract

Previous emotion-regulation research has shown that the late positive potential (LPP) is sensitive to the down-regulation of emotion; however, whether LPP is also sensitive to the up-regulation of emotion remains unclear. The present study examined the description-based reappraisal effects on the up-regulation of positive emotions induced by erotic and neutral images in a Chinese population. Self-reported ratings and event-related potential (ERP) were recorded when subjects viewed pleasant and neutral images, which were shown after either a neutral or positive description. Self-reported results showed that images following positive descriptions were rated as more pleasant compared to images following neutral descriptions. ERP results revealed that the P2, P3, and slow wave (SW) components were larger for erotic pictures than for neutral pictures, while the positive description condition yielded attenuated erotic image-induced P2, P3 and SW and increased SW induced by neutral images. The results demonstrated that description-based reappraisal, as a method of reappraisal, significantly modulates the emotional experience and ERP responses to erotic and neutral images.

## Introduction

The inability to modulate emotions, i.e., dysregulation of emotion, may precipitate affective disorders. Since emotion regulation contributes significantly to mental health, an increasing number of studies have focused on this subject (Taylor and Liberzon, 2007; Amstadter, 2008; Mak et al., 2009). Emotion regulation refers to the process by which individuals regulate the category, timing, experience, and expression of one's emotions (Gross, 1998). In addition, emotion regulation may decrease, maintain, or increase levels of negative and/or positive emotions (Masters, 1991; Parrott and Schulkin, 1993; Langston, 1994). There are two regulation subtypes, i.e., down-regulation and up-regulation, which decrease or increase an emotional experience, respectively. Most previous research has focused on down-regulation, especially the down-regulation of negative emotions (Levesque et al., 2003; Ochsner et al., 2004). For example, using functional magnetic resonance imaging (fMRI), researchers have found that the prefrontal cortex and/or amygdala participate in the down-regulation of negative emotions (Ochsner et al., 2002; Levesque et al., 2003). In addition, event-related potential (ERP) studies have investigated the time course of the down-regulation of emotion processes and have shown that the late positive potential (LPP), an ERP component, is sensitive to the regulation of negative emotions, such that the magnitude of the LPP in response to emotional pictures is significantly attenuated when individuals are instructed to suppress their emotions (Moser et al., 2006; Foti and Hajcak, 2008). Foti and Hajcak (2008) found that both the LPP magnitude and arousal ratings were significantly reduced when unpleasant pictures were described in neutral rather than in negative terms.

To our knowledge, there are limited studies investigating up-regulation, especially the up-regulation of positive emotions. Understanding up-regulation of positive emotions is important for the pleasures of everyday interactions (Ochsner et al., 2004) because the pursuit of happiness may be the eternal theme throughout human life (Kringelbach and Berridge, 2009). For example, an up-regulation strategy could be used either to augment the joy at a wedding or to create a positive emotional experience in response to a previously appraised neutral stimulus (Langston, 1994). Researchers have tried to investigate the underlying mechanism of up-regulation of positive emotions due to the practical and theoretical implications of a positive emotional experience. These studies applied the reappraisal strategy, a form of cognitive strategy that modifies the affective feeling to emotion-eliciting situations by modulating the understanding of these situations. The reappraisal strategy is more effective than the suppression strategy in the down-regulation (Hajcak and Nieuwenhuis, 2006; Goldin et al., 2008) or the up-regulation of negative emotions (Ochsner et al., 2004). Unfortunately, these studies failed to detect significant differences between the up-regulation condition and the control condition regarding positive emotions (Moser et al., 2006; Krompinger et al., 2008).

This study applied the task paradigm of two previous studies (Moser et al., 2006; Krompinger et al., 2008) and modified the paradigm in two aspects. First, it is worth noting that in previous studies which used simple word instructions (e.g., “suppress” or “enhance”) for emotion regulation, the ERP results revealed an attenuated LPP in the down-regulation condition but an unchanged LPP in the up-regulation condition (Moser et al., 2006; Krompinger et al., 2008). Accordingly, we suggest that the presentation of simple wording may not be appropriate for up-regulation. Instead, the current study used a description-based reappraisal strategy, which has been proven to be effective at down-regulating negative emotions and up-regulating neutral emotions (Foti and Hajcak, 2008; Macnamara et al., 2009, 2010).

Second, seeing that the current study was conducted in China, we used erotic pictures as positive stimuli. Compared to Western cultures, Chinese people adhere to more traditional moral values and are consequently less “open” to sexual freedom (Higgins et al., 2002). As a result, Chinese subjects reported less pleasure than Western subjects when viewing the same erotic pictures (Lang et al., 1999; Yen et al., 2010). In our opinion, finding a way to up-regulate Chinese people's positive emotions when facing erotic stimuli would have important implications in improving their quality of life and feeling of well-being.

In the current study to up-regulate emotion in Chinese participants, we selected pictures that depicted erotic couples as the positive stimuli and pictures of household objects as the neutral stimuli. A description-based reappraisal strategy was applied by using a brief neutral or positive description that was presented prior to the emotional pictures. In the regulation condition, the descriptions suggested that the persons in the picture were lovers conforming to traditional morality roles (positive description). Meanwhile, in the control condition, the descriptions indifferently mentioned the impersonal aspects of the pictures (neutral instruction). The present ERP research had three predictions: (1) the description-based reappraisal strategy would successfully raise the levels of pleasant feelings generated by erotic and neutral pictures among Chinese participants; (2) the LPP (P3 and slow wave) would be sensitive to the picture types, such that an increased LPP would be detected when viewing pleasant (erotic) pictures; and (3) the LPP (P3 and slow wave) would be sensitive to the instruction types, such that the LPP magnitude would be different between the up-regulation condition and the control condition. In addition to the LPP, an ERP component that is associated with several psychological processes and the most important ones are motivation and sustained attention (Ibanez et al., 2012). The LPP is enhanced for motivationally relevant stimuli (Schupp et al., 2000, 2004) and is sensitive to cognitive tasks (Polich, 2007; Frühholz et al., 2009; Hurtado et al., 2009; Ibáñez et al., 2011); we also analyzed the frontal P2 component, because previous studies suggested that the P2 was an index of emotional processing (Foti and Hajcak, 2008; Olofsson et al., 2008; Luo et al., 2010). Furthermore, because of well-known gender differences in the processing of erotic stimuli (Lykins et al., 2007), we also analyzed the gender difference in the up-regulation effects and the respective ERP correlates, even though gender differences were not the main emphasis of the present study.

## Materials and methods

### Participants

Forty subjects (20 females) were recruited from either the South China Normal University or Beijing Normal University of China. The average age of subjects was 21.43 years old (*SD* = 2.24). All participants were right-handed and had normal or corrected normal vision. In addition, all subjects were free from any neurological impairment. Subjects were paid for participation and provided written informed consent. The local ethics review board approved this study.

### Stimuli

The stimulus set was composed of 52 neutral (low-arousal) and 52 pleasant (high-arousal) color images. The neutral images (household objects) were selected from the Chinese Affective Picture System (Bai et al., 2005). The procedure of selection of the pleasant pictures was exactly the same as that used in the development of the Chinese affective picture system. All of the pleasant images depicted erotic couples that did not expose their sexual organs. A separate cohort of 35 Chinese participants evaluated the erotic pictures (arousal rating: *M* = 6.02, *SD* = 0.56, valence rating: *M* = 5.88, *SD* = 0.30, *N* = 35). Eight pleasant images(sport) and eight neutral images (antique) were presented as filler materials to keep the participants concentrating on the experiment; however, these filler materials were excluded from the subsequent analysis. Prior to each picture, a brief description of the upcoming picture was presented. There were two types of instructions (i.e., positive and neutral) for each picture. Positive instructions highlighted the positive aspects of the image, whereas neutral instructions described the images in neutral terms. A sample of participants (*N* = 30) evaluated all of the descriptions, and the positive descriptions were markedly more optimistic compared to the neutral descriptions.

A within-subjects 2 (picture type: pleasant and neutral) × 2 (instruction type: positive and neutral) × 2 (gender: males and females) mixed-factors ANOVA included a total of 240 trials. As in previous research (Moser et al., 2006; Krompinger et al., 2008), the 240 trials within the experimental conditions were randomly separated into different blocks, resulting in two pleasant picture blocks (the valence and arousal rating of the pleasant images in the positive and neutral instruction conditions were matched) and two neutral picture blocks (the stimuli in each condition were match as well). To ensure enough time for participants to rest, each block was divided into four sub-blocks, which resulted in a total of 16 sub-blocks. Each sub-block contained 15 pictures (8 positive sub-blocks contained 13 erotic couples pictures and 2 sporting pictures, 8 neutral sub-blocks contained 13 household object pictures and 2 antique pictures). The serial position of the four experimental condition blocks and of the stimuli in each block were counterbalanced across participants.

### Experimental tasks and procedures

Participants sat on a comfortable chair in front of a computer screen, which was located at eye level at a distance of 75 cm. After they finished the informed consent form, participants were attached to an electroencephalograph (EEG) sensor net and given the task instructions. Participants were told that prior to each picture, there would be a brief description about the upcoming picture. Then, participants were instructed to rate the valence of each proceeding picture on a 9-point scale: from 1 (very unpleasant) to 9 (very pleasant).

**Figure 1:**
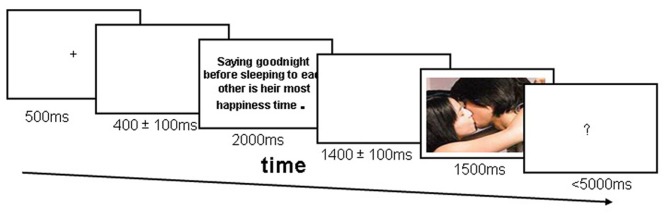
**Experimental design for a single trial.** To give an example, the neutral description for an erotic picture might be “This is a scene of Gaby and Hellen in the room,” the positive description for the same erotic picture might be “No matter where or when, the couple loves each other very much;” the neutral description for a neutral picture (e.g., a computer) might be “This desktop computer belongs to Dike and Amy,” the positive description for the same neutral picture might be “It is the computer that gave them a chance to know each other and fall in love.”

After a practice trial session, the formal experiment commenced, and all participants performed 16 sub-blocks with appropriate breaks. E-prime software was used to control the presentation and timing of all stimuli. As is shown in Figure 1, a fixation mark (+) was presented for 500 ms at the beginning of each trial to orient participants to the center of the screen. The brief description appeared 300–500 ms after the offset of the fixation cross and remained on the screen for 2000 ms. Thereafter, the target picture was presented for 1500 ms after a blank screen (randomized presentation between 1300 ms and 1500 ms). Next a prompt (“?”) appeared in the center of the screen, which cued participants to rate the valence of the picture. After a 1500 ms delay, the next trial began. Color images were 8 × 11 cm, and each picture occupied approximately 6° of the horizontal visual angle and 8.4° of the vertical visual angle (or approximately 8.4° horizontally and 6° vertically).

### EEG recording and data analysis

The Brain Products system (Brain Products GmbH, Munich, Germany) continuously recorded EEG signals from 64 scalp electrodes based on the 10–20 system, with two electrodes placed on the left and right mastoids. Eye blinks and movements were recorded on an electrooculograph (EOG) from four facial electrodes: two approximately 1 cm above and below the participants' left eye, one approximately 1 cm to the left of the left eye, and one approximately 1 cm to the right of the right eye. The EEG was sampled at 500 Hz. All EEG/EOG electrode impedances were below 5 KΩ. Off-line analysis of data was performed using Brain Vision Analyzer software (Brain Products). All data were re-referenced to the average of the two mastoid recordings and band-pass filtered with cutoffs of 0.1 and 30 Hz. The EEG was segmented for each trial, beginning 200 ms before each picture onset (which served as the baseline) and continuing for 1000 ms.

Based upon the suggestions from Krompinger et al. (2008), we measured mean amplitudes in three windows following stimulus onset: 150–200 ms (P2), 330–400 ms (P3), and 400–800 ms (slow wave). For each window, data analysis involved repeated-measure analysis of variance (ANOVA) with the factors picture type (pleasant vs. neutral), instruction type (positive vs. neutral) and gender (male vs. female) as the main factors. Significance level was set at *p* = 0.05 for all analyses. *Post-hoc* testing of significant main effects was conducted using the Bonferroni method. Significant interactions were analyzed using simple-effects models.

## Results

### Self-reported ratings results

Table 1 shows the means and standard deviations for the four experimental conditions. As expected, experienced-pleasure ratings were higher for pleasant (erotic) images than neutral images, *F*_(1, 38)_ = 41.98, *p* < 0.01, indicating a sizable picture-type effect. The main effect of instruction-type was also significant, *F*_(1, 38)_ = 18.35, *p* < 0.01, indicating higher pleasure ratings for positive instructions compared to neutral instructions. The main effect of gender was not significant, *F*_(1, 38)_ = 0.18, *p* = 0.67. There were no other statistically significant effects among these three factors.

**Table 1:** **Results of pleasant ratings**.

**Instruction type**	**Picture type**	***M***		***SD***	
		**Male**	**Female**	**Male**	**Female**
Positive	Pleasant	7.13	6.92	1.02	0.95
	Neutral	5.74	6.26	0.77	1.08
Neutral	Pleasant	6.87	6.44	0.97	1.20
	Neutral	5.79	5.50	0.90	0.66

*Note: It is a 9-point scale: from 1 (very unpleasant) to 9 (very pleasant), which is wildly used by previous study (Lang et al., 1999; Bai et al., 2005)*.

### ERP results

**Figure 2:**
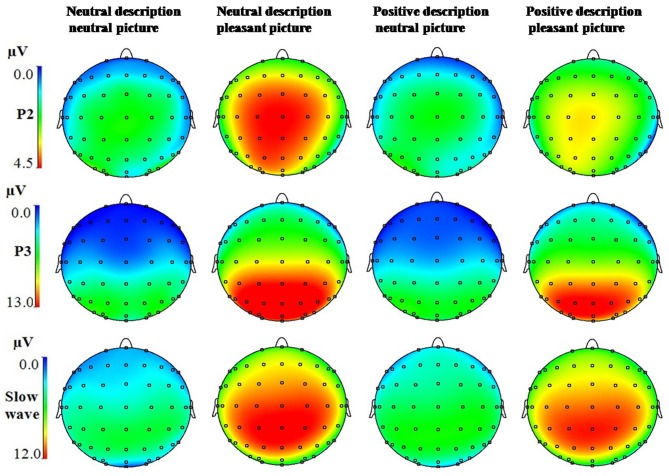
**The topographic map of the P2 (150–200 ms), P3 (330–400 ms) and slow wave (400–800 ms) components for the four conditions**.

Consistent with previous research, P2 was largest at electrode Cz (4.80 μV) in all conditions (see Figure 2). Accordingly, the mean amplitudes of this electrode and eight adjacent electrodes (FCz, FC1, FC2, C1, C2, CPz, CP1, and CP2) were chosen for further analysis. P3 and the slow wave (SW) reached maximum (10.34 μV for P3, and 8.58 μV for slow wave) at electrode Pz, The mean amplitudes of this electrode and 8 adjacent electrodes (CPz, CP1, CP2, POz, PO1, PO2, P1, and P2) were chosen for further analysis.

#### P2 (150–200 ms)

**Figure 3:**
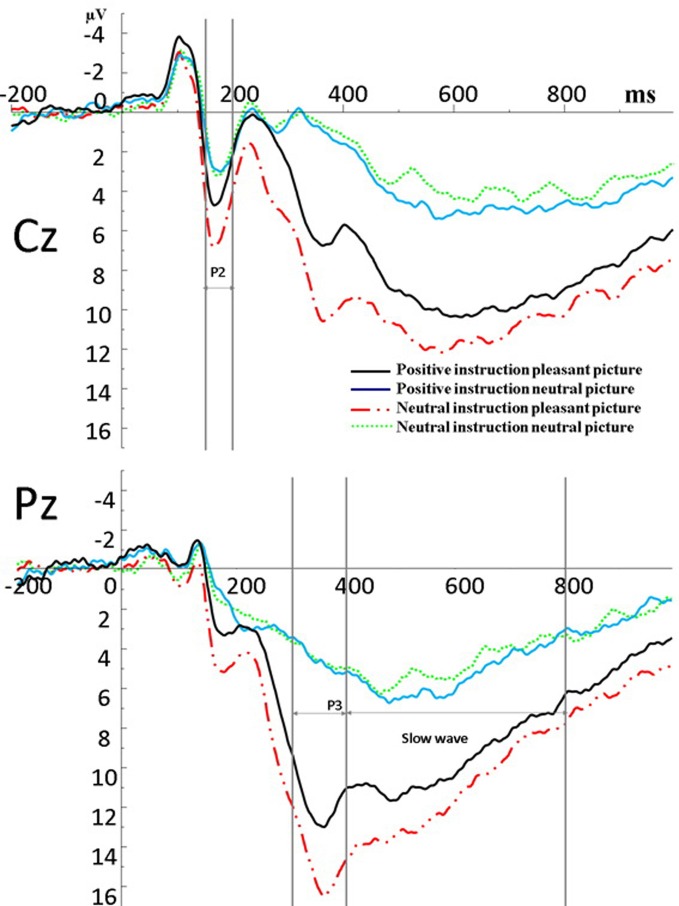
**Stimulus-locked ERPs at Cz and Pz for the four conditions.** The vertical lines at Cz indicate the time windows (150–200 ms) submitted to statistical analysis of the P2 component. The vertical lines at Pz indicate the time windows (330–400 and 400–800 ms) submitted to statistical analysis of the P3 and Slow wave components.

**Figure 4:**
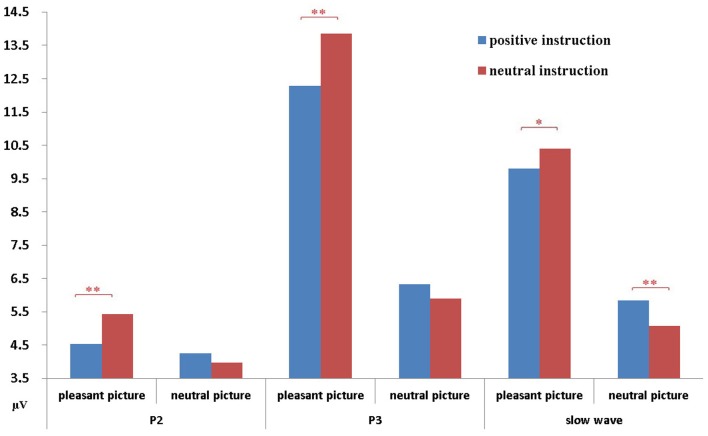
**The effects of instruction-type on P2, P3, and the slow wave component.** Instruction-type had different effects on picture type for P2, P3, and the slow wave components; error bars represent standard errors. ^*^α <0.05, ^**^α<0.01.

Figure 3 presents the mean amplitude for each component in each condition. The amplitude of P2 significantly differed between neutral pictures and pleasant (erotic) pictures, *F*_(1, 38)_ = 7.91, *p* < 0.01, indicating the amplitude of pleasant (erotic) pictures (*M* = 4.97) was significantly larger than neutral pictures (*M* = 4.11). The main effect of instruction-type was not significant, *F*_(1, 38)_ = 2.62, *p* = 0.12. However, an interaction effect between instruction type and picture type was found to be significant, *F*_(1, 38)_ = 9.45, *p* < 0.01. As Figure 4 shows, the simple-effect analysis of this interaction revealed that for the pleasant (erotic) images, the amplitude of P2 evoked by those images following neutral descriptions (*M* = 5.42) was significantly larger than that of pleasant images following positive descriptions (*M* = 4.52), *p* < 0.01, but not significantly different among the neutral images, *p* = 0.25. The main effect of gender was not significant, *F*_(1, 38)_ = 1.48, *p* = 0.23, and gender did not have any significant interactions with the other factors.

#### P3 (330–400 ms)

As expected for the P3 component, there was a significant main effect of picture type, *F*_(1, 38)_ = 141.70, *p* < 0.01, indicating that the amplitude for pleasant (erotic) pictures (*M* = 13.07) was significantly larger than for neutral pictures (*M* = 6.11). There was also a significant main effect of instruction-type, *F*_(1, 38)_ = 9.04, *p* < 0.01. Interestingly, the P3 elicited by pictures in the neutral instruction condition (*M* = 9.88) was significantly larger than in the positive instruction condition (*M* = 9.30). The interaction between picture type and instruction type was also significant, *F*_(1, 38)_ = 14.42, *p* < 0.01. Simple-effect analysis of this interaction revealed that the influence of the instruction type on the P3 associated with pleasant (erotic) images was significant, *p* < 0.01, but not significant on the P3 associated with neutral images, *p* = 0.16 (see Figures 3, 4). The main effect of gender was significant, *F*_(1, 38)_ = 10.72, *p* < 0.01, indicating that females had a greater response (*M* = 4.97) than males (*M* = 4.11) when viewing pictures. However, gender did **not** have any other statistically significant effects or interactions.

#### Slow wave (400–800 ms)

For the SW component, a repeated ANOVA revealed a significant main effect of picture type, *F*_(1, 38)_ = 118.84, *p* < 0.01, such that the amplitude of the SW elicited by pleasant (erotic) pictures (*M* = 10.10) was significantly larger than that elicited by neutral pictures (*M* = 5.46). As a main effect, instruction type failed to reach significance, *F*_(1, 38)_ = 0.16, *p* = 0.69. Picture type and instruction type yielded a significant interaction, *F*_(1, 39)_ = 14.87, *p* < 0.01. Simple-effect analysis of this interaction revealed that for the pleasant (erotic) images, the amplitude of the SW in the positive instruction condition (*M* = 9.80) was significantly smaller than that in the neutral instruction condition (*M* = 10.40), *p* < 0.05. In contrast, the amplitude of the SW in the positive instruction condition (*M* = 5.84) was significantly larger than that in the neutral instruction condition for the pleasant (erotic) images, (*M* = 5.08), *p* < 0.01 (see Figures 3, 4). Gender failed to establish a significant main effect, *F*_(1, 38)_ = 2.67, *p* = 0.11, and it did not have any significant interactions with the other factors.

## Discussion

The present study investigated the effects of description-based reappraisal on the up-regulation of positive emotions induced by erotic or neutral images in a Chinese population. Our goal was to assess the electrophysiological index of positive-emotion enhancement with the application of a description-based reappraisal strategy. Self-reported measures revealed that erotic pictures were rated significantly more positive than neutral pictures, suggesting that erotic pictures induced positive emotions. In addition, pictures following positive instructions were rated more positively than those following neutral instructions, regardless of picture content (erotic or neutral). These results validate an enhancement of positive emotions, indicating that description-based reappraisal is sufficient for positive emotion up-regulation.

In regards to ERP, highly arousing erotic images evoked significantly larger mean amplitudes of P2 and LPP components (including P3 and the slow wave) compared to low-arousal neutral images, a finding that is consistent with prior ERP research (Hajcak and Nieuwenhuis, 2006; Moser et al., 2006; Foti and Hajcak, 2008). These results support the theory that the LPP amplitude is sensitive to the level of emotional arousal (Schupp et al., 2000, 2004; Keil et al., 2001).

Importantly, our data demonstrated that description-based reappraisal regulated the amplitudes of P2 and LPP to the erotic images and the SW response to the neutral images. More specifically, erotic images caused attenuated P2 and LPP responses in the up-regulation condition, while larger P2 and LPP signals were observed in the control condition; neutral images induced an increased SW in the un-regulation condition compared to the control condition. Description-based reappraisal influences participants' emotional experiences by providing a context in which the scene is re-evaluated, and thus affecting the allocation of attentional resources. In our opinion, the results of current study support the existence of the context-dependence effect which has been observed in many domains, such as visual perception, emotion perception and social cognition (Bar, 2004; Barrett et al., 2007; Fedota et al., 2012; Ibañez and Manes, 2012).

In the present study, we investigated the mechanisms of the up-regulation of pleasant emotion with a specific focus on the process of description-based reappraisal regulating pleasant emotions. In our opinion, description-based reappraisal helps an individual maintain a “moderate arousal state.” It is widely recognized that a state of moderate arousal improves memory, while extreme or low arousal impairs it (Seybold, 2007; McMorris et al., 2011). Psychologists have suggested that individuals feel most contentment at the moderate level of arousal (Molinsky, 2007). It is also worth noting that highly pleasurable pictures are usually linked with moderate arousal levels (e.g., in the IAPS, highly pleasurable pictures, such as pictures of babies, had a valence of approximately 8 while their arousal levels were approximately 5, see Lang et al., 1999). Saarni suggests that the outcome of effective (positive) regulation is for an excited organism to return to a state of equilibrium (Saarni, 1997). In other words, positive regulation, which aims to regulate emotion optimistically, including up-regulation of high-arousal positive emotions, down-regulation of highly negative emotions, and up-regulation of low-arousal neutral images, will lead to a moderate arousal state. Numerous findings, including fMRI results, support the idea of the down-regulation of negative emotions to a moderate arousal state. For instance, Ochsner et al. (2004) used a reappraisal strategy to down-regulate highly negative emotions by asking participants to rethink the positive outcomes of negative scenes. The fMRI results revealed decreased activation of the amygdala (Ochsner et al., 2004), which is associated with the state of arousal (Gläscher and Adolphs, 2003). In terms of ERP studies, previous research found that when preceded by neutral descriptions, the amplitude of the LPP elicited by unpleasant images was decreased (Foti and Hajcak, 2008; Macnamara et al., 2009). Another study using moderately arousing pleasant pictures reported that the amplitude of the LPP in the up-regulation condition was smaller (but not significantly) than that observed in response to passive viewing of images (Krompinger et al., 2008). In the present study, highly arousing erotic pictures elicited extremely large LPP signals in the neutral-descriptions condition, demonstrating that the arousal level of the erotic pictures was extremely high, which is consistent with previous research carried out in a sample of Taiwanese (Yen et al., 2010). We suggest that when erotic pictures were reappraised to be more positive, participants would be less aroused to keep a moderate state of arousal rather than becoming more highly aroused.

Another notable finding in the present study is that neutral images induced an increased SW response in the up-regulation condition, which also supported the idea that positive regulation leads to a moderate state of arousal. The neutral images were non-emotional and non-arousing. Therefore, it is understandable that neutral images induced an extremely small SW in the control condition, which is in accordance with prior research (Hajcak and Nieuwenhuis, 2006; Moser et al., 2006; Foti and Hajcak, 2008; Krompinger et al., 2008). When neutral images were guided by positive descriptions in the up-regulation condition, positive emotions (e.g., love, contentment) emerged. Hence, neutral images were experienced as emotional stimuli and therefore induced an increased SW response (see also Macnamara et al., 2009). Nevertheless, it is a pity that we only asked participants to rate the valence of the pictures because we then lack information about the arousal levels of the pictures. Further studies should ask participants to rate the arousal levels of the pictures as well as the valence.

Regarding the influence of gender on emotion processing, self-reported measures failed to find any difference between males and females in the up-regulation of emotions. However, ERP results revealed that P3 was sensitive to gender such that females exhibited a larger P3 than males when processing either erotic or neutral pictures. Similar to previous findings (Whittle et al., 2011), it seems that females were more emotionally reactive than males. ERP results showed that the up-regulation effect was not different between males and females, suggesting that the description-based reappraisal strategy could effectively up-regulate emotion in both genders.

Finally, because the current study focused on whether the up-regulation strategy could produce more positive emotions, we asked the participants to rate the subjective pleasure level of each picture, although the arousal level remained untested. Consequently, it is unclear whether the factor of emotional arousal could account for our results. We admit that this is a limitation, and further studies interested in the potential influence of emotion-regulation instruction on the arousal level of stimuli should take this point into account. Another limitation is that we only used erotic images as pleasant stimuli. Consequently, the question of whether the significant results found in the present study could be applied to other types of pleasant images needs further study.

In summary, the self-reported ratings from our study demonstrated the description-based reappraisal strategy as an effective up-regulator of positive emotions in both genders. We found that the description-based reappraisal regulating the positive emotions induced by erotic images starts at the earlier phase of the ERP (P2, 150–200 ms) and results in decreased ERP responses to the erotic images. In contrast, the up-regulation of positive emotions induced by neutral images starts at the later phase of the ERP (the slow wave, 400–800 ms) and causes a larger SW.

### Conflict of interest statement

The authors declare that the research was conducted in the absence of any commercial or financial relationships that could be construed as a potential conflict of interest.
